# The prognostic role of coeliac node metastasis after resection for distal oesophageal cancer

**DOI:** 10.1038/srep43744

**Published:** 2017-03-03

**Authors:** Martin Rutegård, Pernilla Lagergren, Asif Johar, Ioannis Rouvelas, Jesper Lagergren

**Affiliations:** 1 Department of Molecular Medicine and Surgery, Karolinska Institutet, Karolinska University Hospital, 171 76 Stockholm, Sweden; 2 Department of Surgical and Perioperative Sciences, Umeå University, Umeå, Sweden; 3 Center for Digestive Diseases, Karolinska University Hospital, Division of Surgery, CLINTEC, Karolinska Institutet, Stockholm, Sweden; 4 Division of Cancer Studies, King’s College London, United Kingdom

## Abstract

It is uncertain whether coeliac node metastasis precludes long-term survival in distal oesophageal cancer. This nationwide population-based cohort study included patients who underwent surgical resection for stage III or IV distal oesophageal cancer in 1987–2010 with follow-up until 2014. A minority (17.0%) had neoadjuvant therapy. The prognosis in patients with coeliac node metastasis was compared with patients with no such metastasis and patients with more distant metastasis. Multivariable Cox proportional-hazards regression models provided hazard ratios (HRs) with 95% confidence intervals (CIs) of disease-specific and overall mortality. Among 446 patients, 346 (77.6%) had no coeliac node metastasis, 56 (12.6%) had coeliac node metastasis, and 44 (9.9%) had more distant metastasis. Compared to coeliac node negative patients, coeliac node positive patients were at a 52% increased risk of disease-specific mortality (HR = 1.52, 95% CI 1.10–2.10), while patients with more distant metastasis had a 27% statistically non-significant increase (HR = 1.27, 95% CI 0.88–1.83). Patients with distant metastasis had no increase in disease-specific mortality compared to those with coeliac node metastasis (HR 0.71, 95% CI 0.40–1.27). Thus, patients with distal oesophageal cancer with coeliac node metastasis seem to have a similarly poor survival as patients with more distant metastasis, and thus may not benefit from surgery.

Globally, oesophageal cancer is the 6^th^ and 9^th^ most common cause of death from cancer in men and women, respectively^1^. In the last four decades, a rapid increase in the incidence of oesophageal adenocarcinoma has been witnessed in most Western societies^2^,^3^. As the incidence of oesophageal squamous cell carcinoma has declined, the incidence of adenocarcinoma has surpassed that of squamous cell carcinoma in many Western populations^4^. The majority of patients present with advanced disease, resulting in an overall poor prognosis (<15% 5-year survival)^5^. The curatively intended treatment usually involves extensive surgical resection^5^,^6^, which offers a limited (30–40%) chance of 5-year survival^5^,^6^,^7^. The prognosis is closely linked with the tumour stage at diagnosis, and in patients who undergo surgery, lymph node metastasis status is the strongest prognostic factor. There is a great need for optimised surgical treatment in these patients, and one of the major controversies is the extent of the lymphadenectomy. Recent studies from our group have indicated no prognostic benefit from removing more nodes during oesophagectomy after adjustment for surgeon volume^8^,^9^. Whether the presence of coeliac node metastasis is an indication for surgery carried out with curative intent or palliation alone is a matter of debate. Some surgeons state that coeliac node metastasis indicates generalised disease, not amenable to surgery, while others insist that the involvement of coeliac nodes does not exclude a reasonable chance of cure. This debate prompted the present study, which represents a population-based experience of surgery for distal advanced oesophageal cancer in Sweden. We tested the hypothesis that survival is similarly poor in patients with coeliac node metastasis as in patients with more distant metastasis.

## Methods

### Design

The design of this nationwide Swedish population-based cohort study has been presented in detail elsewhere^8^,^10^. In brief, oesophageal cancer patients were identified using the Swedish Cancer Registry, which is 98% complete for this cancer^11^,^12^. Information about oesophagectomy was derived from the Swedish Patient Registry, which has been nationwide complete from 1987 onwards. The assessment of oesophageal cancer surgery in the Patient Registry has been validated against operation charts (n = 1358), showing a positive predictive value of 99.6%^13^.

Eligible for this study were patients who underwent oesophagectomy for distally located oesophageal cancer of tumour stages III or IV during the period 1^st^ January 1987 to 31^st^ December 2010. These patients were followed up for death or emigration until 30^th^ November 2014. The study exposure was the presence or absence of coeliac node metastasis. The main study outcome was disease-specific mortality, while overall mortality was the secondary outcome. Data on prognostic factors were collected to allow adjustment for potential confounding.

### Approvals

The study was approved by the Regional Ethical Review Board in Stockholm. All methods were carried out in accordance with relevant guidelines and regulations. Individual informed consent was not acquired as this is not necessary for this type of study (based on registry data and medical records) according to Swedish law.

### Data Collection

Data on surgical procedures (including type, calendar year, and hospital) were retrieved from the operation charts. Comorbidity was ascertained through the Swedish Patient Registry^14^, and defined according to the most recent update of the well-validated Charlson comorbidity system^15^. Information about tumour stage, tumour histology, macroscopic radicality, and neoadjuvant therapy was collected from the histopathological records of the resected specimens. Date of death and causes of death were retrieved from the Swedish Causes of Death Registry, which is >99% complete.

### Exposure and Outcome Definitions

Tumour stage was classified according to the Union Internationale Contre le Cancer, using the 6^th^ edition of the tumour-node-metastasis (TNM) system^16^. We used this edition to investigate the impact of coeliac node involvement in distal oesophageal cancer. In this context, the 6^th^ edition defines stage III cancer as at least T3 with regional lymph node involvement or T4, while stage IVA is defined as tumour with coeliac node metastasis, and stage IVB is defined as tumour with more distant spread. For the purpose of the present study, we determined these stages as “no coeliac node metastasis” (6^th^ edition: stage III), “coeliac node metastasis” (6^th^ edition: stage IVA) and “distant metastasis” (6^th^ edition: stage IVB), respectively. The assessment of coeliac lymph node metastasis was made possible by sending coeliac nodes separately or marked in the en-bloc specimen by one of the surgeons. Patients with distant metastasis who underwent surgery had the operation only because the metastasis was not identified prior to surgery. Disease-specific mortality was defined by a cause of death including oesophageal cancer in the Causes of Death Registry. Overall mortality indicated death independent of the cause.

### Statistical Analysis

Mortality was assessed up until 5 years after surgery. First, the Kaplan-Meier method was used, stratified by absence or presence of coeliac node metastasis as well as more distant spread, and tested for statistical significance with the log-rank test. Second, multivariable Cox proportional-hazards regression was employed to calculate hazard ratios (HRs) with 95% confidence intervals (CIs) adjusted for potential confounders. Three predefined regression models were used: unadjusted, basic, and full. The basic model comprised of age (categorised into <65, 65–75 or >75 years), sex (male or female) and Charlson comorbidity score (0, 1 or ≥2). The full model further included resection margin status (without [R0] or with [R1-R2] tumour involved margins), tumour histology (adenocarcinoma or squamous cell carcinoma), neoadjuvant treatment (yes or no), calendar period of surgery (1987–1994, 1995–2002 or 2003–2010), and hospital volume (>9 or ≤9 operations per year). Hospital volume was dichotomised using the median value during the entire study period. In sensitivity analyses, we excluded postoperative deaths, defined as death within 90 days of surgery. The proportional-hazards assumption was tested by the introduction of time-varying interactions with the model covariates. Since missing data were rare, we used complete case analysis. All p-values were two-tailed and considered statistically significant when below 0.05. The statistical software SAS 9.4 (SAS Institute, Cary, NC) and STATA version 12 (StataCorp, Houston, Texas) were used for all analyses.

## Results

### Patients

The original cohort included 1,820 patients who had undergone resection for oesophageal cancer. Of these, 446 patients with a distal tumour of stage III or IV that was either coeliac node negative (n = 346; 77.6%), coeliac node positive (n = 56; 12.6%), or metastasised beyond the coeliac nodes (n = 44; 9.9%), remained for final analysis. Clinical characteristics of these three exposure groups are presented in Table 1. Most patients (83.0%) had no neoadjuvant therapy in all groups. Female sex, involved resection margins, and postoperative mortality were overrepresented in the distant metastasis groups.

### Survival Rates

The overall 5-year survival in the coeliac node negative, coeliac node positive and distant metastasis groups amounted to 9.5%, 5.4% and 0.0%, respectively. Median survival in these groups was 10.5, 6.3 and 7.5 months, respectively. The corresponding 5-year disease-free survival rates were 11.9%, 6.0% and 0.0%, while the median survival was 9.3, 6.0 and 7.4 months, respectively.

### Survival Curves

**Figure 1:**
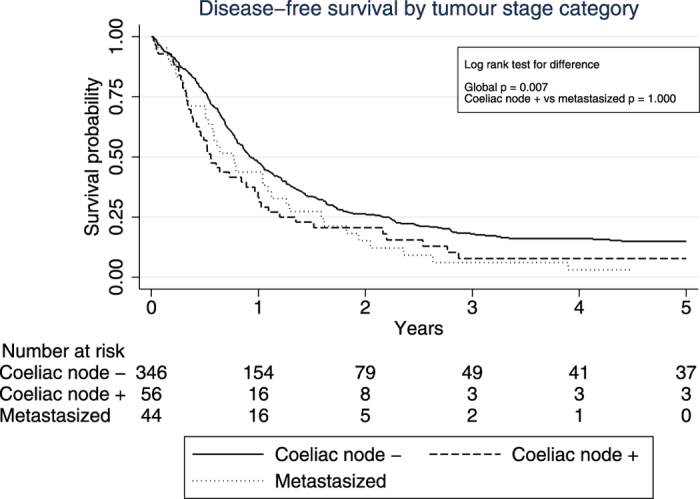
Kaplan-Meier curves depicting disease-free survival in 446 patients operated for advanced distal oesophageal cancer, stratified by the following metastasis categories: coeliac node negative, coeliac node positive and distant metastasis.

**Figure 2:**
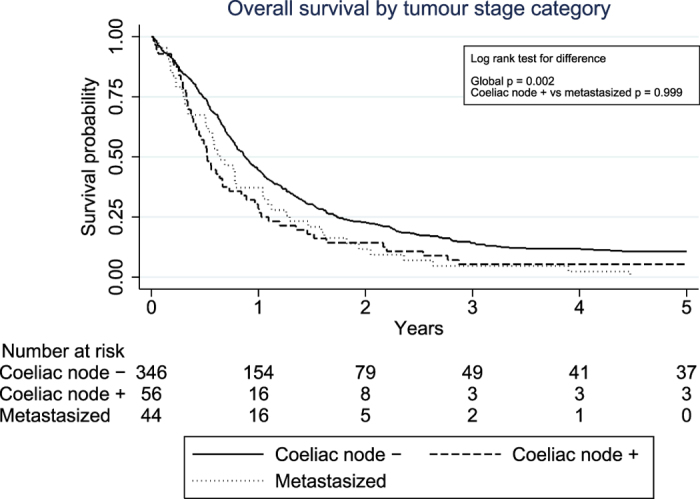
Kaplan-Meier curves displaying overall survival in 446 patients operated for advanced distal oesophageal cancer, stratified by the following metastasis categories: coeliac node negative, coeliac node positive and distant metastasis.

Kaplan-Meier curves depicting the disease-free and overall survival in the three groups are displayed in Figs 1 and 2. The log-rank tests showed differences in disease-free and overall survival between coeliac node negative patients, patients with coeliac node metastasis and patients with more distant spread (p = 0.007 and p = 0.003 for disease-free and overall survival, respectively), while no such differences were found between coeliac node positive patients and those with more distant spread (p = 1.000 and p = 0.891 for disease-free and overall survival, respectively).

#### Relative Risk of Disease-Specific Mortality

The results of the Cox regression models are presented in Table 2. In comparison with coeliac node negative patients, patients with coeliac node metastasis had an increased risk of disease-specific mortality (fully adjusted HR = 1.52, 95% CI 1.10–2.10), while the corresponding risk increase was 27% in patients with more distant metastasis (fully adjusted HR = 1.27, 95% CI 0.88–1.83). Patients with distant spread had no increased disease-specific mortality compared to those with coeliac node metastasis (fully adjusted HR = 0.71, 95% CI 0.40–1.27).

#### Relative Risk of Overall Mortality

When compared to coeliac node negative patients, node positive patients had an increased overall risk of mortality (fully adjusted HR = 1.54, 95% CI 1.14–2.09). The HR was also statistically significantly increased for patients with a more distant spread (fully adjusted HR = 1.42, 95% CI 1.01–1.98). Patients with distant metastasis had a similar risk of overall mortality as patients with coeliac node metastasis (fully adjusted HR = 0.93, 95% CI 0.55–1.59).

#### Additional Analyses

The sensitivity analyses excluding mortality during the initial 90 days of surgery did not alter the results (data not shown). There was no evidence of violation of the proportional-hazards assumption in any of the regression models.

## Discussion

In this study of surgically resected distal advanced oesophageal cancers, the long-term prognosis was similar in patients with coeliac node metastasis and in those with more distant spread, and considerably worse compared to patients without coeliac node involvement.

Among the strengths of the current study are the population-based design, large sample size, complete and long follow-up of all participants, and the adjustment for key prognostic factors. Limitations include a lack of data on potential postoperative (adjuvant) treatment. However, the use of postoperative therapy was negligible in Sweden during the study period. Data were lacking on comorbidity diagnoses only recorded prior to the initiation of nationwide registration by the Swedish Patient Registry in 1987. However, important comorbidities should have been registered at the point of hospitalisation for oesophagectomy, and these were adjusted for in the analyses. We had accurate data on postoperative tumour stage, but there is a lack of data on preoperative staging and staging techniques used. Moreover, we were unable to take into account different operative strategies such as en-bloc resections or separate coeliac node retrieval. This potentially leads to tumour stage misclassification preoperatively and postoperatively, as different methods and strategies across hospitals concerning radiology, operative technique and histopathology may yield varying rates of correctness in the assessment of coeliac node status, and especially the presence of distant metastasis at the time of surgery. To add, during most of the study period, neoadjuvant therapy was not common, and results may therefore be difficult to generalise to an era when such therapy is generally recommended. It is possible that involved coeliac nodes can respond well to oncological therapy in some patients and still offer them a chance of cure. Finally, the long study period itself, though necessary to accumulate enough statistical power for the study question, poses potential problems due to changes in the clinical management over time. However, we adjusted for calendar period, which should reduce any such confounding.

Other population-based studies are scarce. In a single-centre study of 144 patients with predominantly distally located oesophageal or gastro-oesophageal junctional tumours subject to neoadjuvant chemoradiotherapy followed by surgery, patients with coeliac node metastasis had a 5-year survival of 13%, while it was 36% in patients without such metastases^17^. In a study of 67 patients with surgically resected tumours, the 5-year survival was 27.0% and 19.5% in M1a and M1b disease in the 6^th^ TNM classification system, respectively^18^. However, only a third of these patients had coeliac node metastasis, while the remainder represented recurrent node metastasis, which limits comparison to the present study. In another single-centre study of 82 patients who underwent surgery without neoadjuvant therapy, the 5-year survival was 8% and 5% for M1a and M1b tumours, respectively^19^. The same research group published surgical results following neoadjuvant therapy, achieving a median survival of 26 months, and a 5-year survival of 19% in a group preoperatively suspected of coeliac lymph node involvement^20^. However, only 52% in this patient group proved to have involved lymph nodes according to pathological status, which may explain the differences in survival compared to the current study. In an additional surgical series of 310 patients, coeliac node negative and coeliac node positive patients experienced similar survival^21^. This contradicts our findings, but the differences in design and our ability to adjust for all relevant confounders might explain the divergent findings.

Further well-designed studies are required to establish whether coeliac node metastasis should exclude patients from curatively intended therapy. There is also a need to objectively verify the presence of such metastasis if they will determine the clinical decision-making, e.g. by endoscopic ultrasonography-guided fine needle aspiration or laparoscopy with biopsies^22^.

In conclusion, this population-based and nationwide Swedish study indicates that patients with distal oesophageal cancer with coeliac node metastasis may not benefit from surgical resection as survival seems to be similarly as poor as in patients with more distant spread. Accurate preoperative staging of coeliac node metastases might improve patient selection for surgery. However, since this cohort includes only a limited proportion of patients treated with neoadjuvant therapy the findings might not be fully applicable to preoperatively treated patients.

## Additional Information

**How to cite this article:** Rutegård, M. *et al*. The prognostic role of coeliac node metastasis after resection for distal oesophageal cancer. *Sci. Rep.* **7**, 43744; doi: 10.1038/srep43744 (2017).

**Publisher's note:** Springer Nature remains neutral with regard to jurisdictional claims in published maps and institutional affiliations.

## Figures and Tables

**Table 1:** Clinical characteristics in 446 patients resected for advanced distal oesophageal cancer (coeliac node negative, coeliac node positive and those with distant metastasis) in Sweden in 1987–2010.

Characteristic	Tumour stage category			
	All (N = 446)	Coeliac node negative (N = 346)	Coeliac node positive (N = 56)	Distant metastasis (N = 44)
	Number (%)	Number (%)	Number (%)	Number (%)
**Age**				
<65 years	215 (48.2)	165 (47.7)	32 (57.1)	18 (40.9)
65–75 years	159 (35.7)	122 (35.3)	19 (33.9)	18 (40.9)
>75 years	72 (16.1)	59 (17.1)	5 (8.9)	8 (18.2)
**Sex**				
Male	354 (79.4)	277 (80.1)	48 (85.7)	29 (65.9)
Female	92 (20.6)	69 (19.9)	8 (14.3)	15 (34.1)
**Charlson comorbidity index**				
0	0 (0.0)	0 (0.0)	0 (0.0)	0 (0.0)
1	198 (44.4)	154 (44.5)	25 (44.6)	19 (43.2)
≥2	248 (55.6)	192 (55.5)	31 (55.4)	25 (56.8)
**Histology**				
Adenocarcinoma	249 (55.8)	198 (57.2)	28 (50.0)	23 (52.3)
Squamous cell carcinoma	197 (44.2)	148 (42.8)	28 (50.0)	21 (47.7)
**Resection margin status**				
R0	294 (65.9)	241 (69.7)	39 (69.6)	14 (31.8)
R1-R2	122 (27.4)	79 (22.8)	16 (28.6)	27 (61.4)
*Missing*	30 (6.7)	26 (7.5)	1 (1.8)	3 (6.8)
**Neoadjuvant therapy**				
Yes	76 (17.0)	56 (16.2)	10 (17.9)	10 (22.7)
No	370 (83.0)	290 (83.8)	46 (82.1)	34 (77.3)
**Calendar period**				
1987–1994	105 (23.5)	81 (23.4)	12 (21.4)	12 (27.3)
1995–2002	102 (22.9)	74 (21.4)	19 (33.9)	9 (20.5)
2003–2010	239 (53.6)	191 (55.2)	25 (44.6)	23 (52.3)
**Hospital volume**				
>9 operations per year	208 (46.6)	162 (46.8)	24 (42.9)	22 (50.0)
≤9 operations per year	238 (53.4)	184 (53.2)	32 (57.1)	22 (50.0)
**Postoperative mortality ≤90 days**				
No	387 (86.8)	304 (87.9)	49 (87.6)	34 (77.3)
Yes	59 (13.2)	42 (12.1)	7 (12.5)	10 (22.7)

**Table 2:** Disease-specific and overall mortality in 446 patients resected for advanced distal oesophageal cancer, expressed as hazard ratios (HRs) with 95% confidence intervals (CIs).

Tumour stage category	Patients	Unadjusted	*P value*	Basic*		Full†	
	*Number*	*HR 95% CI*		*HR 95% CI*	*P value*	*HR 95% CI*	*P value*
Disease-specific mortality							
Coeliac node negative	346	1.00 (reference)		1.00 (reference)		1.00 (reference)	
Coeliac node positive	56	1.51 (1.11–2.06)	0.009	1.56 (1.13–2.12)	0.006	1.52 (1.10–2.10)	0.011
Distant metastasis	44	1.43 (1.02–2.02)	0.041	1.47 (1.04–2.08)	0.032	1.27 (0.88–1.83)	0.19
Coeliac node positive	56	1.00 (reference)		1.00 (reference)		1.00 (reference)	
Distant metastasis	44	0.97 (0.63–1.50)	0.195	0.96 (0.60–1.53)	0.342	0.71 (0.40–1.27)	0.506
Overall mortality							
Coeliac node negative	346	1.00 (reference)		1.00 (reference)		1.00 (reference)	
Coeliac node positive	56	1.52 (1.13–2.03)	0.005	1.54 (1.15–2.06)	0.004	1.54 (1.14–2.09)	0.005
Distant metastasis	44	1.53 (1.11–2.09)	0.009	1.60 (1.16–2.21)	0.004	1.42 (1.01–1.98)	0.041
Coeliac node positive	56	1.00 (reference)		1.00 (reference)		1.00 (reference)	
Distant metastasis	44	1.02 (0.69–1.53)	0.908	1.09 (0.71–1.69)	0.686	0.93 (0.55–1.59)	0.801

^*^Adjusted for age, sex, and comorbidity.

^†^Adjusted for age, sex, comorbidity, histology, resection margin status, neoadjuvant therapy, calendar period, and hospital volume.
